# Leukotoxin (LtxA/Leukothera) induces ATP expulsion via pannexin-1 channels and subsequent cell death in malignant lymphocytes

**DOI:** 10.1038/s41598-021-97545-4

**Published:** 2021-09-10

**Authors:** Derek J. Prince, Deendayal Patel, Scott C. Kachlany

**Affiliations:** 1 Department of Oral Biology, Rutgers School of Dental Medicine, Newark, NJ 07103 USA; 2 Gibraltar Laboratories, Inc., Fairfield, NJ 07004 USA

**Keywords:** Bacterial toxins, Bacteriology, Cell death

## Abstract

Leukotoxin (LtxA) (Trade name, Leukothera) is a protein that is secreted from the oral bacterium *Aggregatibacter actinomycetemcomitans*, which targets and kills activated white blood cells (WBCs) by binding to lymphocyte function associated antigen-1 (LFA-1). Interaction between LtxA and Jurkat T-cells results in cell death and is characterized by increased intracellular Ca^2+^, activation of caspases, clustering of LtxA and LFA-1 within lipid rafts, and involvement of the Fas death receptor. Here, we show that LtxA can kill malignant lymphocytes via apoptotic and necrotic forms of cell death. We show that LtxA causes activation of caspases and PARP, cleavage of pannexin-1 (Panx1) channels, and expulsion of ATP, ultimately leading to cell death via apoptosis and necrosis. CRISPR-Cas9 mediated knockout (K/O) of Panx1 in Jurkat cells prevented ATP expulsion and resulted in resistance to LtxA for both apoptotic and necrotic forms of death. Resistance to necrosis could only be overcome when supplementing LtxA with endogenous ATP (bzATP). The combination of LtxA and bzATP promoted only necrosis, as no Panx1 K/O cells stained positive for phosphatidylserine (PS) exposure following the combined treatment. Inhibition of LtxA/bzATP-induced necrosis was possible when pretreating Jurkat cells with oATP, a P2X_7_R antagonist. Similarly, blockage of P2X_7_Rs with oATP prevented the intracellular mobilization of Ca^2+^, an important early step in LtxA induced cell death. We show that LtxA is able to kill malignant lymphocytes through an apoptotic death pathway which is potentially linked to a Panx1/P2X_7_R mediated necrotic form of death. Thus, inhibition of ATP release appears to significantly delay the onset of LtxA induced apoptosis while completely disabling the necrotic death pathway in T-lymphocytes, demonstrating the crucial role of ATP release in LtxA-mediated cell death.

## Introduction

*Aggregatibacter actinomycetemcomitans* (A. actinomycetemcomitans) is a Gram-negative oral bacterium found in the oral flora of healthy individuals and patients with aggressive periodontitis. The bacterium produces several virulence factors such as leukotoxin (LtxA), cytolethal distending toxin, immunosuppression factors, lipopolysaccharides, surface antigens, and heat shock proteins, among others, to promote disease in the oral cavity such as aggressive periodontal diseases^1^. Perhaps the most important virulence factor produced by *A. actinomycetemcomitans* is that of LtxA, a 113 kilodalton (kDa) protein strategically secreted to suppress the host immune response to promote the pathogenicity of *A. actinomycetemcomitans*^2,3^. Immune suppression is achieved as LtxA specifically targets and kills activated white blood cells (WBCs) by binding to lymphocyte function-associated antigen-1 (LFA-1). LFA-1 is a β2 integrin [α (CD11a) and β (CD18)] expressed on hematopoietic cells^3^. The primary role of the integrin is to promote leukocyte adhesion through interaction with intercellular adhesion molecules (ICAMs), which can lead to cellular migration and activation^4,5^. In a cellular resting state, LFA-1 is configured in a bent or inactive affinity. Only when activation stimuli (e.g. chemokines and cytokines) are sensed do LFA-1 presenting cells express LFA-1 in its extended or “activated” state^6^. It is this state that initiates the pro-inflammatory/migratory response. Therefore, activation of LFA-1 plays a role in the migration of immune cells to the appropriate tissue in order to establish an inflammatory response and promote diapedesis, as the ability of immune cells to penetrate into tissues depends on activated LFA-1^6,7^. For this reason, LFA-1 is an intriguing therapeutic option for targeting certain white blood cell diseases and inflammatory disorders. In fact, targeting LFA-1 presenting cells with LtxA has already demonstrated significant therapeutic potential in a variety of autoimmune/inflammatory conditions^8–10^. Moreover, it has been observed that LFA-1 is upregulated in many leukemias and lymphomas^11–13^. LtxA has also proven to be an effective single or combination use agent to induce rapid apoptosis in a wide variety of leukemia and lymphoma cell types^4,14,15^. In addition, LtxA has shown targeted activity and safety in mouse models for leukemia and lymphoma^4,9,16^. Therefore, the specific targeting and killing of LFA-1 expressing cells remains an intriguing option for the treatment of hematologic malignancies.

In monocytes, LtxA initiates a lysosomal mediated death pathway where LtxA delivers LFA-1 to lysosomes, disrupting lysosomal integrity and inducing cell death^17^. This was determined via the cytosolic detection of lysosomal-associated membrane protein 1 (LAMP-1) and cathepsin D following LtxA treatment, an indication of lysosomal lysis. However, when this pathway was studied in a lymphocytic leukemia cell model, this lysosomal mediated death mechanism was not observed^4^. Furthermore, Fagerberg et al. shows that an additional mechanism involving ATP release may also be involved in cell death^18^. Instead, it has been established that malignant lymphocytes undergo a LtxA-mediated cell death mechanism that requires the death receptor Fas (CD95)^19^. Using Jurkat cells, our lab has shown that LtxA-mediated cell death requires the death receptor Fas and caspase-8 but not Fas Ligand (Fas-L), implying a non-canonical caspase 8/Fas mediated death pathway^4,19^. However, the precise mechanisms leading up to this pathway and the downstream cascades remain elusive and require further investigation.

In the present work, we sought to better understand the mechanisms of LtxA-mediated cell death of malignant lymphocytes. We report that immediately following LtxA exposure, Jurkat lymphocytes expel ATP via Panx1 channels and the expelled ATP goes on to activate purinergic receptor P2X_7_ (P2X_7_R). As a result, the expelled ATP and activation of P2X_7_R allows the cell to proceed with the mobilization of intracellular Ca^2+^, caspase activation, and PARP cleavage, all of which are key events required for the LtxA-mediated cell death of Jurkat cells.

## Results

### LtxA causes ATP release and cell death (apoptosis) in Jurkat cells

**Figure 1:**
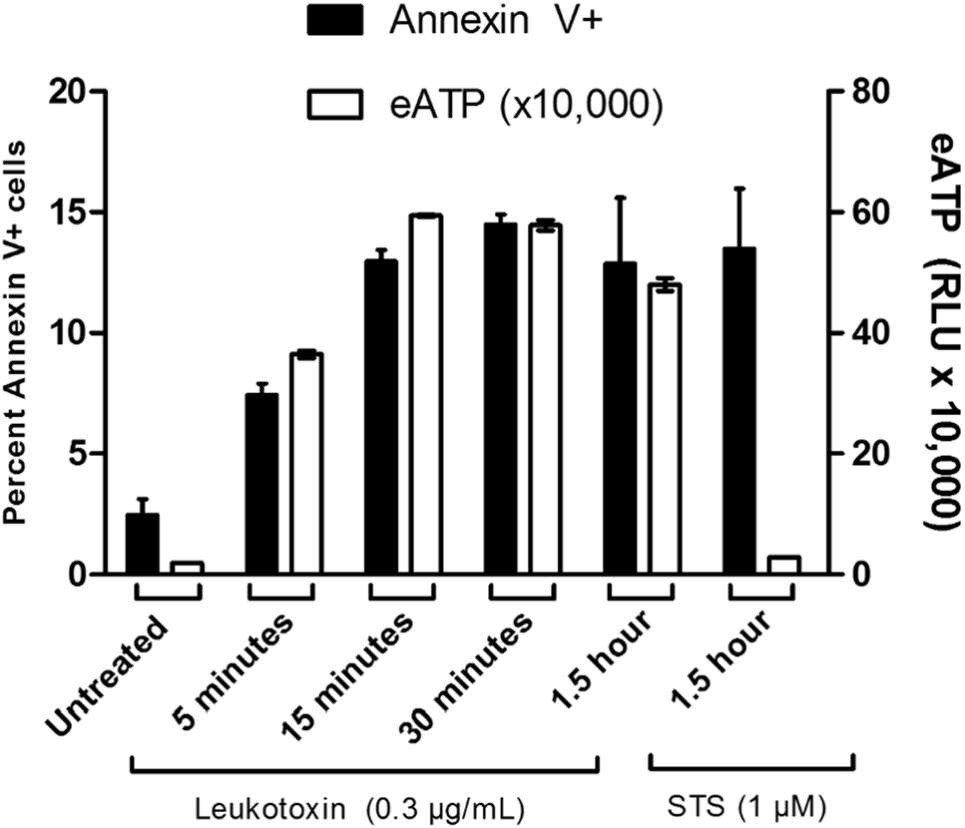
ATP is expelled from Jurkat cells following LtxA treatment. Wild-type Jurkat cells were left untreated or treated with 0.3 µg/mL of LtxA for varying time points or with 1 µM of STS for 1.5 h. Apoptosis was assessed via flow cytometry using the Guava Nexin kit by EMD Millipore through detection of Annexin V. Extracellular ATP (eATP) was measured by immediately spinning down the cells following treatment and measuring the total amount of ATP within cellular supernatants. Apoptosis is expressed as the percentage of gated cells staining positive for Annexin V and eATP is expressed as the percentage of ATP mediated luminescence within sample supernatants compared to the untreated cell control supernatant.

In response to certain cytotoxic and chemotherapeutic agents, transformed lymphocytes have been observed to rapidly (within seconds to minutes) expel ATP in a controlled manner^20–23^. To determine if LtxA causes rapid release of ATP in Jurkat cells, we treated cells with the protein and then measured extracellular ATP. We found that treatment with LtxA (0.3 µg/mL) induces the rapid release of ATP from dying malignant lymphocytes in a time-dependent manner (Fig. 1). STS, a well-studied apoptosis inducing compound in Jurkat cells, did not cause an increase in extracellular ATP.

### LtxA mediated ATP release is not a result of membrane lysis

**Figure 2:**
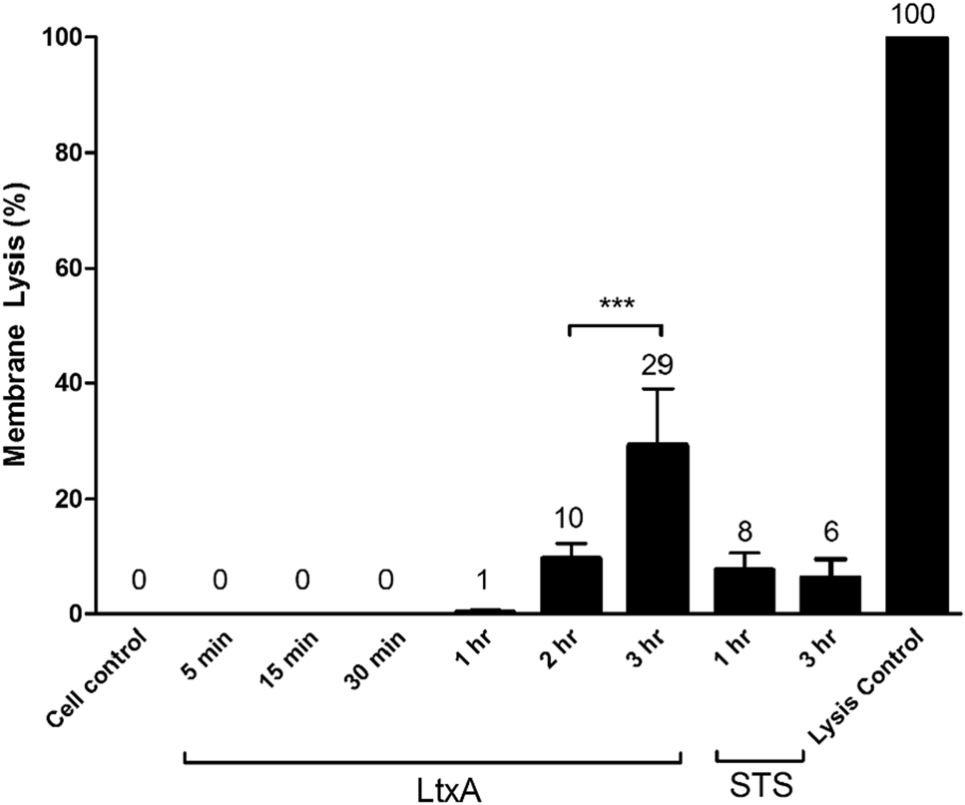
LtxA induced membrane lysis is not the cause of ATP expulsion. Wild-type Jurkat cells were treated with 0.3 µg/mL of LtxA for varying time points or with 1 µM of STS for 1 and 3 h. Membrane lysis was assessed using the Cytotox-ONE Homogenous Membrane Integrity Assay from Promega. Membrane lysis is expressed as a percentage of lactate dehydrogenase (LDH) relative to the control cells.

The controlled secretion of ATP from dying cells is a known mechanism in certain forms of cell death^22^. To determine if ATP was being released from LtxA treated Jurkat cells in a controlled manner, membrane integrity assays were performed. Following treatment with LtxA, membrane lysis was not observed at the same time points where the maximum early release of ATP occurred (15–30 min). We observed that notable membrane lysis does not occur until 2–3 h post LtxA treatment (Fig. 2). Thus, the release of ATP precedes the onset of membrane disruption.

### Panx1 and P2X_7_R protein expression is present in wild-type Jurkat cells

**Figure 3:**
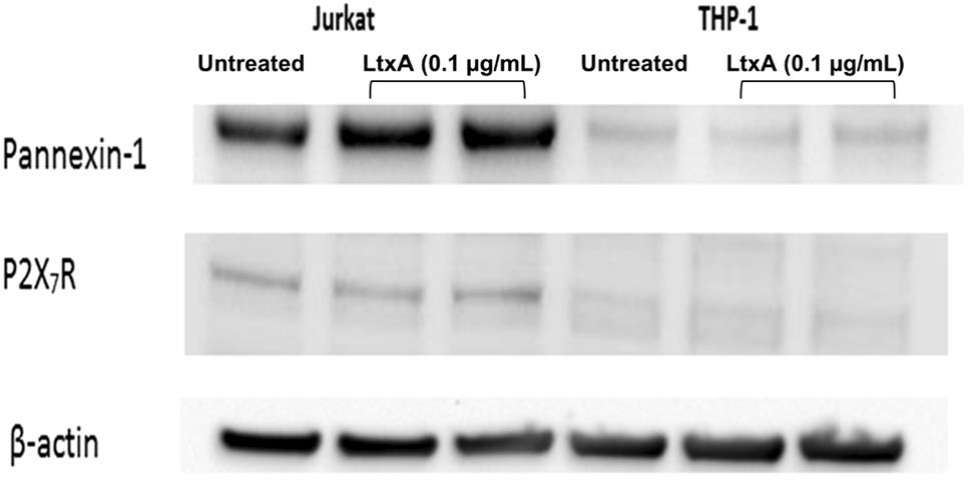
Panx1 and P2X_7_R expression in Jurkat and THP-1 cells. Wild-type Jurkat and THP-1 cells were left untreated or treated with 0.1 µg/mL of LtxA for 1 h. Western blots were performed to determine the expression of Panx1 and P2X_7_R protein content. LtxA treatments are shown in duplicate in lanes 2 and 3 and 5 and 6. β-Actin was used as the loading control. Uncropped gels are presented in Supplementary Figures S1–S3.

Since their discovery in the early 2000s, the pannexin families of membrane pore forming proteins have been increasingly linked to the controlled release of ATP from a broad range of cell types^24^. To test the hypothesis that pannexin proteins could play a role in LtxA-mediated ATP release in Jurkat cells, we performed western blot analysis. We found that Jurkat cells express relatively high concentrations of Panx1 protein expression when compared to a malignant monocytic cell line (Fig. 3). Jurkat cells also express P2X_7_, but at much lower levels than Panx1. Treatment of Jurkat cells with low concentrations of LtxA (0.1 µg/mL) did not increase the expression of Panx1 or P2X_7_R when compared to the cell control. This data indicates that Panx1 is expressed and could play an important signaling role in LtxA mediated cell death of Jurkat cells.

### Generation of a Jurkat Panx1 knockout cell line using CRISPR/Cas9

**Figure 4:**
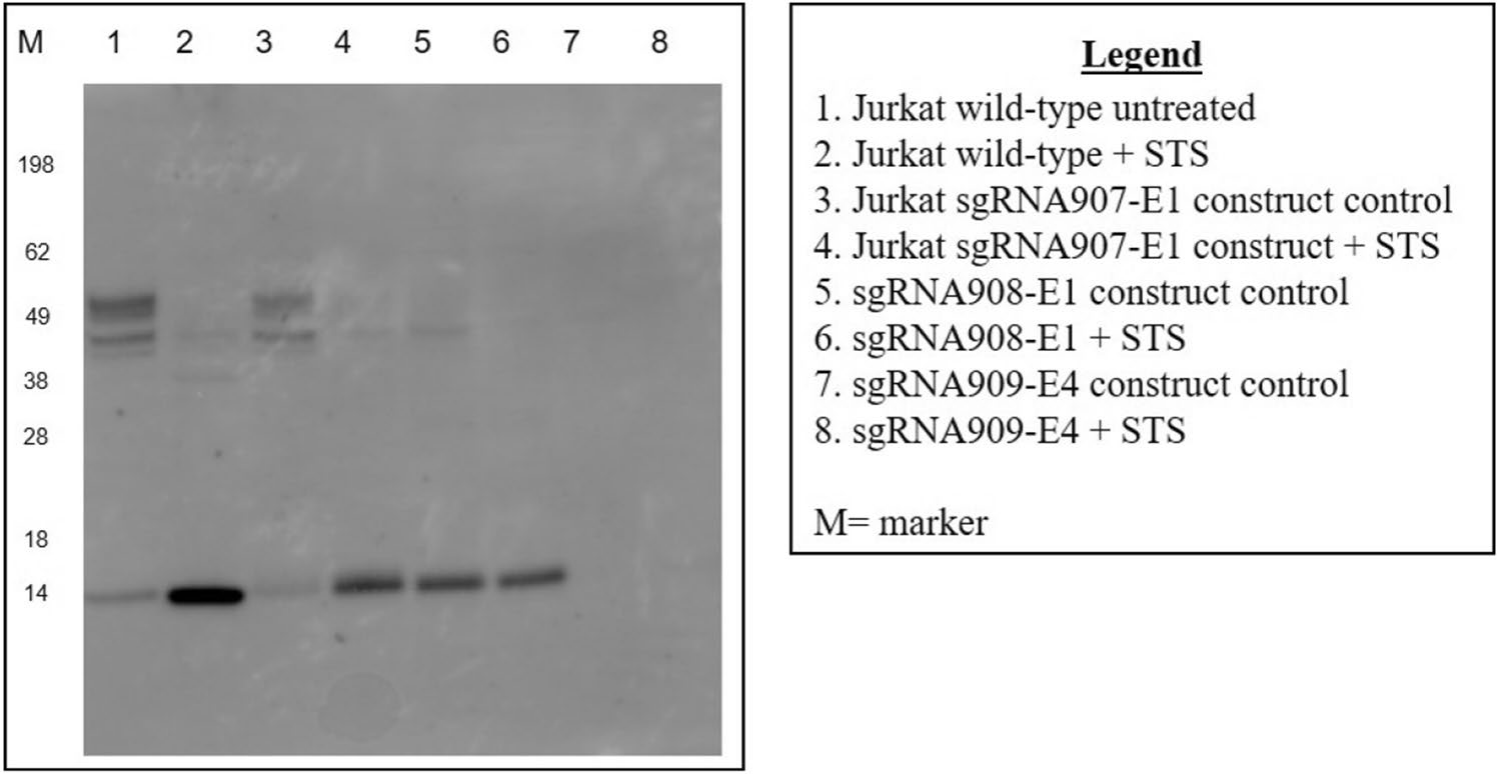
Western blot analysis of Panx1 protein K/O in Jurkat cells. Jurkat cells stably expressing Cas9 were transduced with three different sgRNA’s each targeting a different exon sequence of the Panx1 gene: sgRNA907 (exon 1), sgRNA908 (exon 2), and sgRNA909 (exon 4). Analysis was performed with cells that were successfully transduced and that were expanded through the usage of selection media and single cell isolation/cloning. Western blot analysis was performed to determine which sgRNA construct resulted in the most effective knock down of Panx1 total protein expression. Where indicated, cells were treated with staurosporin (STS) at a concentration of 1 µM for 3 h. STS, known to mediate the cleavage of Panx1, was included to ensure that the assay can detect and that the cells are responsive to a known cell death inducing agent by detecting cleaved forms of Panx1.The lower band represents cleaved Panx1. β-Actin was used as the loading control. The uncropped β-actin gel is presented in Supplementary Figure S4.

Previous studies have reported the importance of Panx1 not only as a membrane channel but as a mediator of inflammation and cell death via its release of ATP^25^. To fully understand the implication of Panx1 as it relates to LtxA mediated cell death of Jurkat cells, a Panx1 knockout was generated using CRISPR/Cas9 methodology. Western blot analysis was performed to determine which sgRNA construct produced the most effective knockout of Panx1 as it relates to total protein expression. Three total sgRNAs were analyzed via transduction. Of the three, sgRNA909 (against Exon 4) completely knocked out Panx1 expression in Jurkat cells (Fig. 4; Lanes 7 and 8) as no Panx1 protein bands were observed at 45 kDa (full product) or 14 kDa (cleaved product). Thus, using this lentiviral based transduction approach; we were able to generate a Jurkat cell line with no detectable expression of any isoforms of the Panx1 protein (Fig. 4). Where indicated, cells were treated with staurosporin (STS) at a concentration of 1 µM for 3 h. β-actin was used as the loading control.

### Panx1 knockout cells exhibit significantly reduced levels of LtxA induced eATP and apoptosis

**Figure 5:**
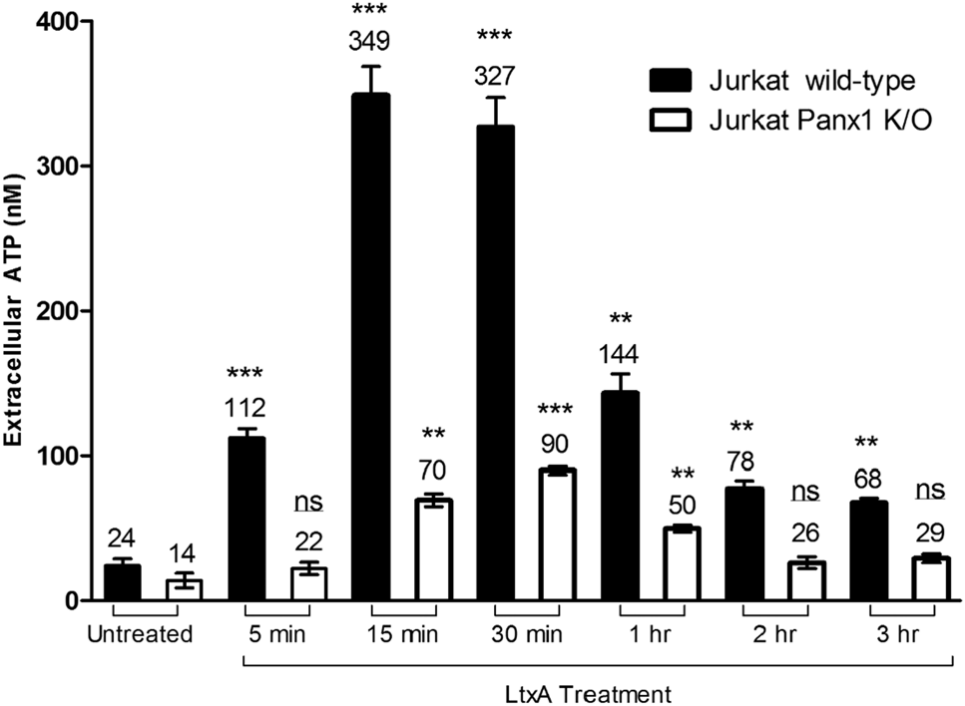
LtxA induced expulsion of ATP from Jurkat wild-type and Panx1 K/O cells. Expulsion of ATP was determined by collecting cellular supernatants of wild-type and Panx1 K/O Jurkat cells following treatments with LtxA or STS and measuring ATP content. Cells were treated with LtxA at a concentration of 0.3 µg/mL. ATP quantification was performed using a 5-point standard curve.

We hypothesized that Panx1 channels could be responsible for the early and controlled release of ATP from LtxA treated Jurkat cells. To determine this, extracellular ATP levels of LtxA treated wild-type and Panx1 K/O Jurkat cells were measured. Knockout of the Panx1 channel resulted in significantly less LtxA-induced expulsion of ATP compared to wild-type Jurkat cells (Fig. 5).

**Figure 6:**
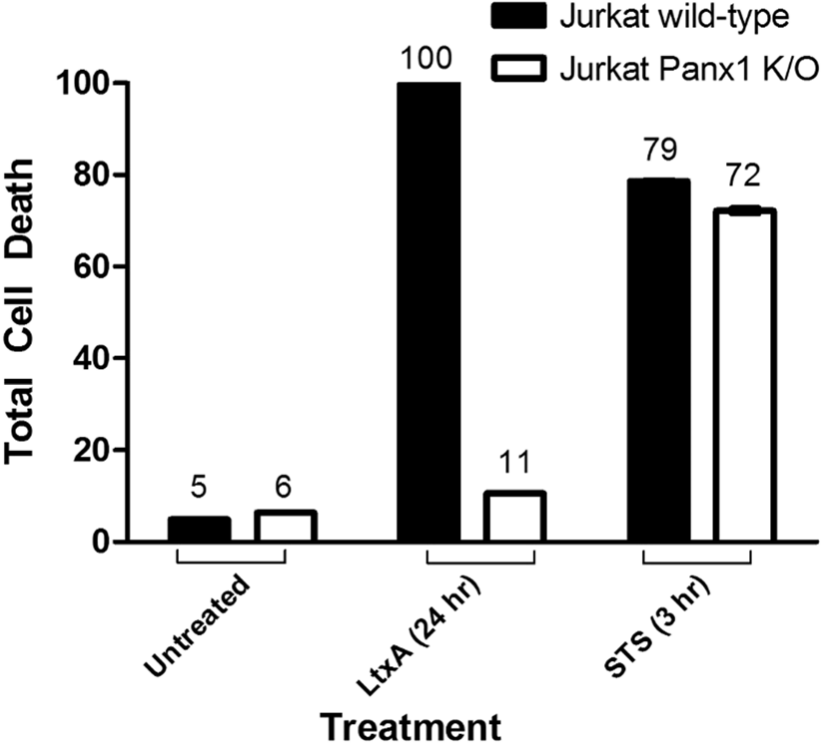
Panx1 K/O cells have reduced sensitivity to LtxA at concentrations of 0.3 µg/mL for as long as 24 h. Jurkat wild-type and Panx1 K/O cells were treated with LtxA for 24 h. Following treatment, Jurkat wild-type cells stained over 100% positive for Annexin while Panx1 K/O cells showed Annexin staining similar to that of untreated control cells. To assess whether the appropriate apoptotic machinery was functioning properly in Panx1 K/O cells, STS was used as a positive control. Both Jurkat wild-type and Panx1 K/O cells showed similar Annexin staining patterns following treatment with STS. Where indicated, cells were treated with LtxA at a concentration of 0.3 µg/mL for 24 h or with staurosporin (STS) at a concentration of 1 µM for 3 h.

**Figure 7:**
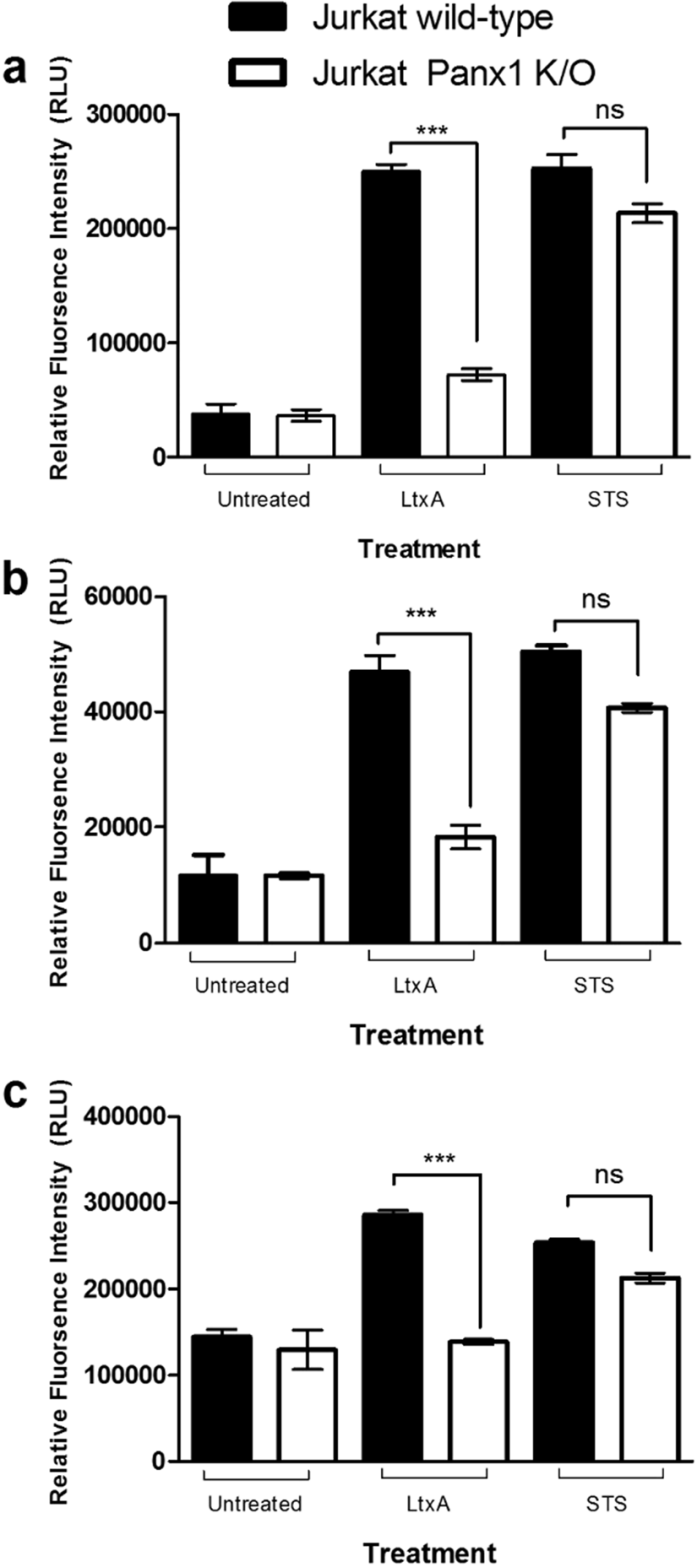
Caspase activation is inhibited in Panx1 K/O cells treated with LtxA. Jurkat wild-type and Panx1 K/O cells were treated with LtxA or STS for 3 h. Cells were then measured for caspase cleavage for caspases 3/7 (**a**), 8 (**b**), and 9 (**c**) using the Caspase-Glo luminescent based microtiter assay from Promega. STS served as a positive control. ***p < 0.0001.

**Figure 8:**
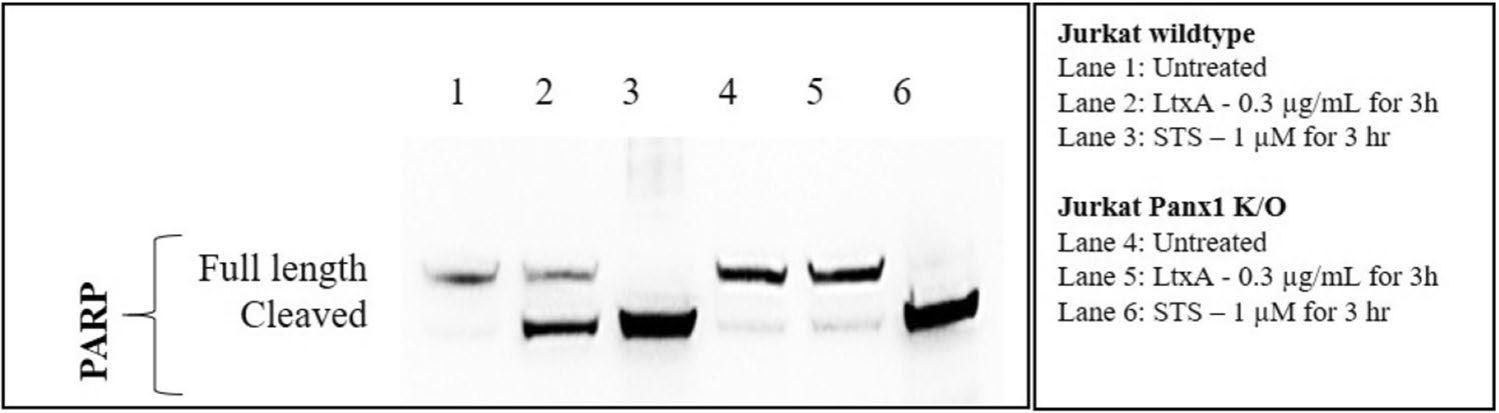
LtxA induced PARP cleavage in Jurkat Wildtype and Panx1 K/O cells. Wild-type and Panx1 K/O Jurkat cells were left untreated or treated with 0.3 µg/mL of LtxA or 1 µM STS for 3 h. Western blots were performed to determine the expression of PARP cleavage. While PARP cleavage was detected in LtxA and STS treated Jurkat wild-type cells and in STS treated Panx-1 K/O cells, no PARP cleavage was detected in LtxA treated Panx-1 K/O cells. β-Actin was used as the loading control (data not shown). The uncropped gel showing full-length and cleaved PARP is presented in Supplementary Figure S5.

To determine if Panx1 channels were linked to LtxA mediated cell death, several cell death assays were performed using Jurkat wild-type and Panx1 K/O cells. When LtxA was added to Panx1 K/O cells, apoptosis and caspase activation were similar to that of the untreated cellular control (Figs. 6, 7). Additionally, LtxA treatment of Panx1 K/O cells resulted in the inhibition of PARP cleavage (an indication of apoptosis) (Lane 5), similar to that of untreated cellular controls (Fig. 8).

### LtxA induces two distinct death pathways in Jurkat cells

**Figure 9:**
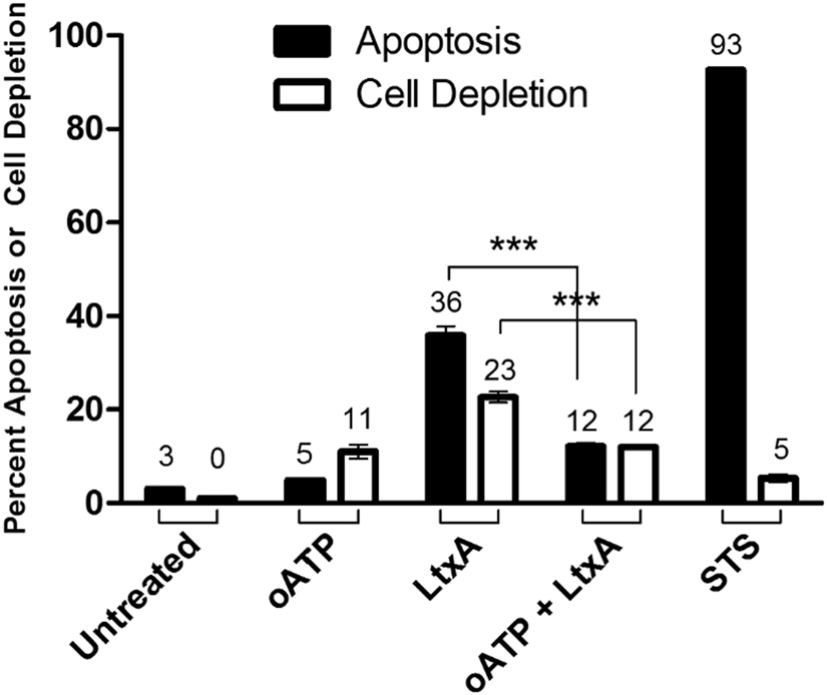
LtxA induces two distinct death pathways in Jurkat cells. Jurkat wild-type cells were treated with LtxA for 3 h with or without oATP. Without oATP, LtxA induces a distinct apoptotic population (Annexin V+) as well as a lytic population (cellular depletion). When Jurkat cells are pre-treated with oATP prior to LtxA treatment, the apoptotic population is decreased and the lytic population is inhibited.

Recent studies have implicated a link between ATP and P2X_7_Rs in apoptotic and necrotic forms of cell death^26^. For this reason, in addition to our assessment of apoptosis, we decided to also measure LtxA mediated cellular depletion (necrosis). To assess the precise role of P2X_7_R as a potential mediator of necrotic cell death, oxidized ATP (oATP) was used. oATP is an irreversible inhibitor of macrophage purinergic receptors that works by covalently modifying the receptors nucleotide binding proteins^27^. Blockage of P2X_7_R with oATP completely inhibited the ability of LtxA to initiate the cellular depletion pathway from proceeding (Fig. 9). In addition, while the typical apoptotic mode of cell death was not completely suppressed, it was significantly decreased (Fig. 9). It was determined that LtxA induces two distinct death pathways: (1) apoptosis and (2) a P2X_7_R mediated form of necrosis (Fig. 9).

### Exogenous ATP promotes the cellular depletion pathway

**Figure 10:**
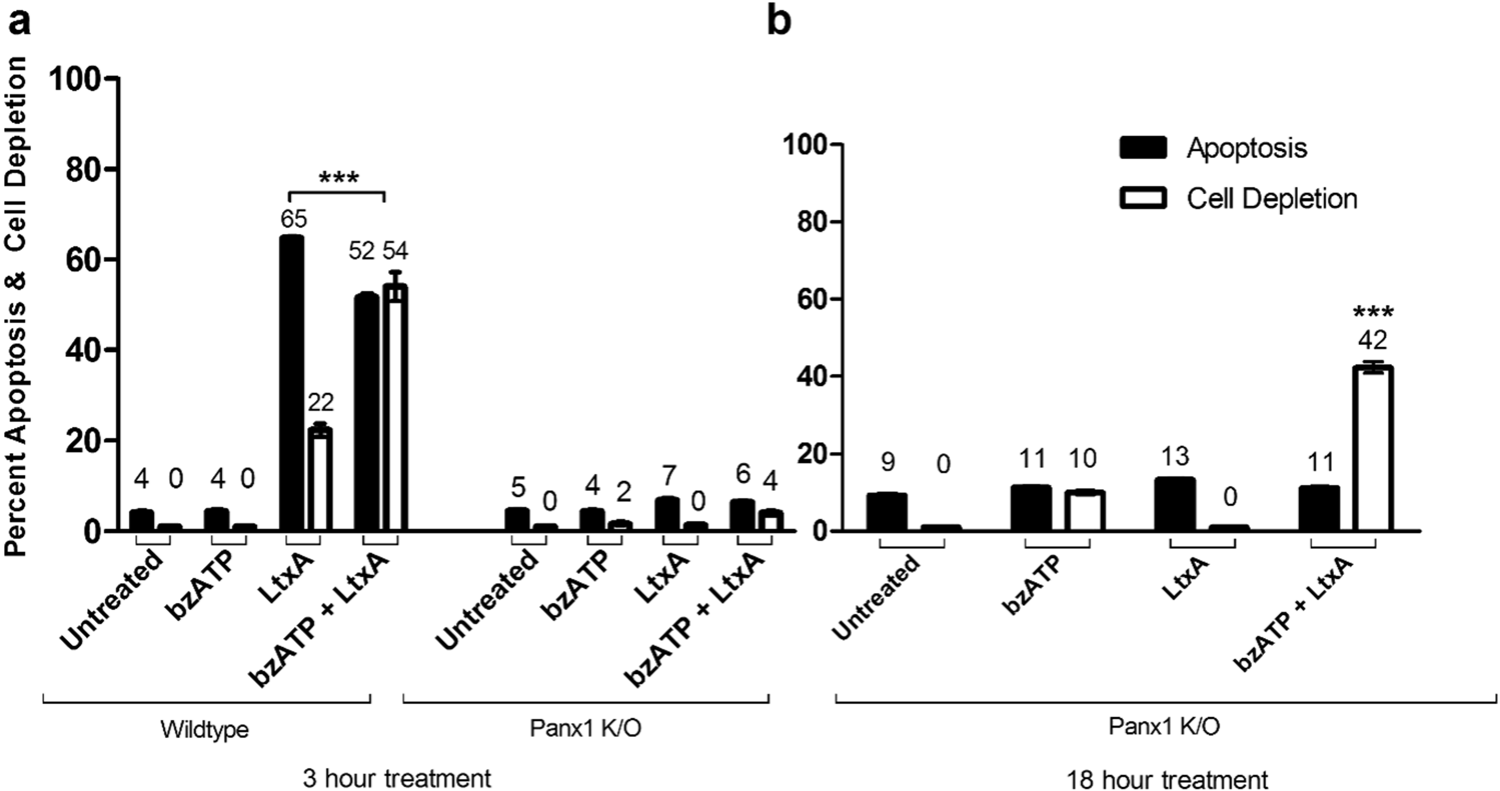
Addition of bzATP promotes LtxA mediated lysis in Jurkat wild-type and Panx1 K/O cells. Jurkat wild-type and Panx1 K/O cells were treated with LtxA with or without bzATP for 3 or 18 h. In Jurkat wild-type cells, the addition of bzATP with LtxA promoted a cytolytic pathway independent of apoptosis in 3 h (**a**). Panx1 K/O cells remained resistant to LtxA and bzATP + LtxA induced apoptosis and lysis for 3 h. When Panx1 K/O cells were stimulated with bzATP and LtxA overnight (**b**), a cytolytic pathway was observed but apoptosis remained deficient.

To further explore the role of ATP in LtxA mediated cell death, LtxA treatments were supplemented with exogenous ATP (bzATP) and apoptosis and cellular depletion were measured in both Jurkat wild-type and Panx1 K/O cells. Following LtxA stimulation of Jurkat wild-type cells, a clear distinction between the apoptotic and cellular depletion pathways is observed after 3 h (Fig. 10a). When LtxA was then supplemented with bzATP, the cellular depletion pathway was drastically increased (Fig. 10a). LtxA alone or in combination with bzATP did not promote apoptosis or cellular depletion in Panx1 K/O cells after 3 h of treatment (Fig. 10a). To determine if the resistance to cell death observed in the K/O cells could be overcome, bzATP stimulation was increased from 3 to 18 h. By increasing the bzATP treatment time and supplementing the treatment with LtxA, promotion of only the cellular depletion pathway in Panx1 K/O cells was observed (Fig. 10b).

### Blockage of P2X_7_R with oATP prevents intracellular Ca^2+^ mobilization in LtxA treated Jurkat cells

**Figure 11:**
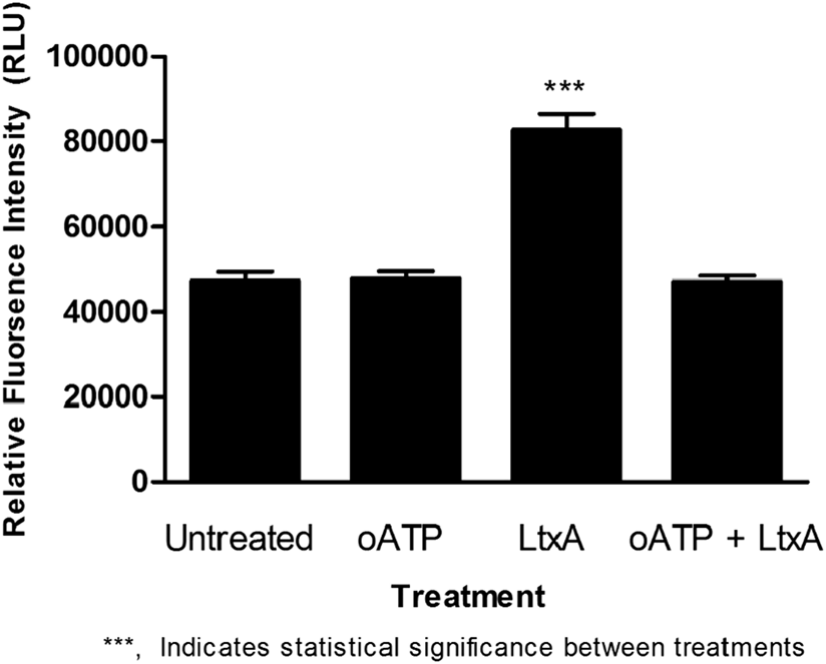
Intracellular Ca^2+^ mobilization of LtxA treated Jurkat cells. Jurkat cells were treated with LtxA alone or pre-treated with oATP following LtxA treatment and assessed for intracellular Ca^2+^ mobilization using a fluorescent based assay. Preventing the binding of ATP to P2X_7_R using oATP reduced intracellular Ca^2+^ mobilization to that of untreated cell control levels. Where indicated, cells were pretreated with oATP at a concentration of 600 µM for 1 h prior to LtxA treatment. LtxA was used at a concentration of 0.3 µg/mL for 30 min. ***p < 0.001.

Mobilization of intracellular Ca^2+^ is one of the earliest and most important LtxA mediated events responsible for the cell death of Jurkat cells^28,29^. To determine if extracellular ATP plays a role in intracellular Ca^2+^ mobilization, the primary binding partner of ATP (P2X_7_R) was blocked with oATP. We demonstrate that cytosolic Ca^2+^ mobilization was decreased to levels similar to that of untreated control cells when Jurkat cells antagonized with oATP were subsequently treated with LtxA (Fig. 11).

### The role of ATP in LtxA mediated cell death

The proposed model describes the molecular sequence of events carried out during LtxA mediated cell death of Jurkat cells. LtxA first interacts with susceptible cells by binding to LFA-1 which is believed to be coupled to the Fas death receptor. This binding and interaction of LFA-1 to the membrane is then thought to initiate the controlled expulsion of ATP via Panx1 channels. The release of ATP then triggers intracellular Ca^2+^ mobilization, clustering of LFA-1 into lipid rafts, and subsequent cell death via apoptosis and necrosis. We additionally propose that the elevations in intracellular Ca^2+^ are dependent on the interaction between ATP and P2X_7_R.

## Discussion

Extracellular ATP (eATP) has been shown to promote a variety of responses in T cells including CD27, CD23, and CD26L shedding, secretion of IL-2 and IFN-γ, cell proliferation, and antigen presentation^30^. Apoptotic cells have also been observed to release ATP and UTP (uridine-5′-triphosphate) at early stages of cell death via Panx1 channels^21^. In this regard, the release of ATP from dying cells has been shown to promote the recruitment of leukocytes to the site of injury^25^. Studies with Jurkat lymphocytes and mouse thymocytes demonstrated caspase-3-dependent activation of Panx1 channels and consequent ATP release in response to the Fas-triggered induction of the extrinsic apoptotic cascade^20^. Knowing that LtxA triggers caspase activation and subsequent Fas mediated cell death in Jurkat cells, we investigated whether Panx1 channels were responsible for the release of ATP and if this contributed to the cell death pathway(s) that we observed.

Following treatment with LtxA, we observed that Jurkat cell lymphocytes cleave caspases and PARP and stain positive for Annexin V (apoptotic marker) (Figs. 7, 8, 9). Despite being marked positive for apoptosis, we also observed that these LtxA treated cells also displayed relatively high levels of eATP compared to cells treated with staurosporin, an inducer of apoptosis (Fig. 1). We sought to better understand how this ATP was being utilized and its role in LtxA mediated cell death.

We determined that ATP was being expelled from cells as a result of LtxA treatment and that the expulsion of ATP is an early event that is not a result of membrane disruption or lysis (Fig. 2). This is of interest because along with the over expression of LFA-1, leukemic leukocytes have been observed to overexpress Panx1 channels^20–22,25,30^. In fact, in response to certain cytotoxic agents, transformed lymphocytes have been observed to expel ATP in a controlled manner^20–22^.

As seen in Fig. 3, we were able to verify the presence of Panx1 in Jurkat cells via western blot analysis. Pretreating Jurkat cells with LtxA concentrations of 0.1 µg/mL for 1 h had no noticeable impact on the amount of protein observed, indicating that LtxA treatment does not appear to upregulate the production of Panx1 protein. Since Panx1 channels have been strongly associated with P2X_7_Rs, we also assessed cell lysates for the presence of P2X_7_R protein (Fig. 3). We observed that THP-1 cells displayed a Panx1 expression band of a much lower intensity when compared to the same population of Jurkat cells. This is important to note because while LtxA triggers ATP release and increased intracellular Ca^2+^ concentrations in THP-1 cells, the actual release of ATP from these cells appears to proceed with mechanisms independent of Panx1 channels^18^. Taken together, we wished to know if the controlled expulsion of ATP was the result of Panx1 signaling and whether P2X_7_R expression also played a role in the LtxA mediated cell death of Jurkat cells.

To determine the potential role of Panx1 in the LtxA induced expulsion of ATP in Jurkat cells, CRISPR/Cas9 methodology was used to generate a Panx 1 K/O cell line (Fig. 4). We determined that LtxA mediated expulsion of ATP was drastically reduced when Panx1 was knocked out (Fig. 5). While complete inhibition of eATP was not observed in Panx1 K/O cells, the released ATP is most likely the result of alternative release mechanisms engaging to compensate for the lack of Panx1.

In addition to the significant reduction of LtxA induced eATP, Panx1 K/O cells were also significantly more resistant to LtxA as cell death following LtxA treatments were similar to that of untreated control cells (Fig. 6). It is important to note that Panx1 K/O cells continued to be susceptible to cell death via staurosporin treatment, indicating that the molecular machinery responsible for initiating and carrying out the extrinsic apoptotic death pathway was not affected in Panx1 K/O cells (Fig. 6, STS control). To corroborate the LtxA resistance data generated through the Annexin V assay, caspase activation assays were also performed. As shown in Fig. 7, K/O of Panx1 reduced LtxA mediated caspase activation (-3/-7, -8, and -9) to control levels while having no effect on the activation of caspases via staurosporin. Further, the cleavage of PARP following treatment with LtxA was also reduced in Panx1 K/O cells (Fig. 8).

With the significant reductions in LtxA mediated cell death following Panx1 K/O, we examined whether Fas or LFA-1 surface expression was affected, as these two receptors are known to be critical for LtxA induced cell death of Jurkat cells^19,31^. Through flow cytometry, it was determined that there was no decrease in LFA-1 or Fas surface expression as the result of our CRISPR/Cas9 mediated K/O of Panx1 (data not shown). This indicates that there is a close connection between Panx1, ATP, PARP cleavage, and caspase activation in relation to LtxA induced cell death of Jurkat cells.

It is known that the activation of P2X_7_R may lead to cytotoxicity and act in a way to upregulate cell death in response to pathological insults^32^. During pro-inflammatory responses and tissue injury, release of ATP into the extracellular environment has shown to activate P2X_7_Rs, resulting in apoptotic and necrotic forms of cell death^26,33^. In murine thymocytes, ATP-mediated P2X_7_R activation leads to death via both caspase-dependent apoptosis and necrosis/lysis, even though necrotic cell death is predominant^26^. Additionally, P2X_7_Rs are involved in many types of leukemias, as ATP-induced activation of P2X_7_R has been implicated to promote a diverse array of signaling events including apoptosis of certain types of leukemic B- and T-cells^34–39^. It has also been demonstrated that the Fas-L mediated death of Jurkat cells requires an ATP-dependent cross talk between Fas and P2X_7_R^35^. The involvement of P2X_7_Rs in LtxA treated macrophages has also been suggested, as macrophages and lymphocytes pre-treated with oATP, a P2X_7_R antagonist, were less susceptible to cell death following certain treatments, such as Fas-L^35,37,38,40^. Since LtxA specifically binds to LFA-1, and LFA-1 appears to colocalize with Fas, it is possible that LtxA is initiating a similar P2X_7_R-mediated response. For this reason, it was important to also investigate the potential involvement of this receptor.

To understand the involvement of P2X_7_Rs, the receptor was physically blocked using oATP prior to LtxA treatment. oATP is an irreversible inhibitor of the macrophage purinergic receptor that works by covalently modifying the receptor’s nucleotide binding proteins, preventing the binding of ATP/eATP^27^. Since previous studies indicated that stimulation of P2X_7_R via eATP could lead to both apoptotic and necrotic forms of cell death, we measured cellular depletion in addition to apoptosis. We determined that LtxA induces two distinct death pathways: (1) apoptosis and (2) a form of necrosis (Fig. 9). When P2X_7_R was blocked with oATP, and the cells were subsequently treated with LtxA, the apoptotic pathway was decreased while cellular depletion was completely inhibited (Fig. 9). While we did not expect pretreatment with oATP to affect apoptosis as strongly as it did, we believe that the reason for the decrease in apoptosis following oATP treatment is due to a potential link between P2X_7_R and Panx1^26^. It is likely that the suppression of P2X_7_R via oATP has a functional effect on Panx1 thereby disrupting ATP release and suppressing or delaying the apoptotic pathway. This data supports the role of P2X_7_R contributing to both the apoptotic and necrotic forms of LtxA induced cell death. Specifically, LtxA induced eATP and activation of P2X_7_R appears to be important with respect to the rate at which apoptosis can initiate and required for the necrotic cell death pathway.

To better understand the potential contribution of P2X_7_R as it relates to the two LtxA initiated death pathways, we investigated as to whether an environment high in eATP was contributing to either of the death pathways. To determine this, LtxA was supplemented with exogenous ATP (bzATP), and apoptosis and cellular depletion were measured in both Jurkat wild-type and Panx1 K/O cells. Based on other reports noting the involvement of exogenous ATP in cellular lysis^41^, and with the knowledge that LtxA initiates apoptotic and necrotic mediated death pathways, we expected that the addition of bzATP to both Jurkat wild-type and Panx1 K/O cells would result in activation of P2X_7_Rs and subsequent cytolysis (cell depletion). Following LtxA stimulation of Jurkat wild-type cells, a clear distinction between the apoptotic and cellular depletion pathways was observed (Figs. 9, 10). When LtxA was then supplemented with bzATP, the cellular depletion pathway was drastically increased (Fig. 10a). This increase in the cellular depletion pathway strongly hints at the combination of LtxA and bzATP promoting a necrotic mechanism of death. Interestingly, Panx1 K/O cells did not react to combinations of LtxA and bzATP as expected (Fig. 10a). We attribute this to the fact that Panx1 K/O cells do not expel ATP at the level of the wild-type cells, nor do they die via traditional caspase mediated death mechanisms following the LtxA concentrations and times studied here. Therefore, these cells are presumably less susceptible to LtxA and bzATP mediated death mechanisms. To determine if the defect could be overcome, bzATP stimulation was increased from 3 to 18 h. By increasing the bzATP treatment time and supplementing the treatment with LtxA, we were able to promote the cellular depletion pathway in Panx1 K/O cells (Fig. 10b). We note that there was still little effect on apoptosis despite the increased exposure time of LtxA and bzATP. There are two possible explanations for this observation. The first is that Panx1 functionality is required in promoting LtxA induced apoptosis and the second is that it is known that overtime LtxA can cause membrane damage. Together, this data further supports that LtxA works in combination with ATP to promote apoptotic and lytic modes of cell death in Jurkat cells. In addition to Panx1 playing a role in the initiation of apoptosis, it also appears to be required for ATP expulsion, stimulation of P2X_7_Rs, and ultimately, cytolysis.

It is understood that the influx of intracellular Ca^2+^ is one of the earliest events known to be important for LtxA mediated cell death^28,29^. In fact, Fong et al. demonstrated that LtxA induces an LFA-1 independent elevation of cytosolic Ca^2+^ in Jurkat cells^29^. This early event leads to the subsequent activation of a cascade of events including calpain activation, talin cleavage, and mobilization and clustering of LFA-1 within lipid rafts along the plasma membrane^29^. We report here that an additional early event is that of the expulsion of ATP, and when compromised, alters how the cell responds to LtxA and subsequent cell death. Shown in Figs. 1, 2, and 5, we demonstrate how LtxA expels ATP from susceptible Jurkat cells early and in a controlled manner. Given the early and important changes mediated by LtxA induced Ca^2+^ mobilization, we investigated whether expelled ATP played a role in the ability for cells to mobilize Ca^2+^.

We discovered that when blocking Jurkat P2X_7_Rs with oATP and subsequently treating the cells with LtxA, cytosolic Ca^2+^ mobilization was decreased to levels of untreated control cells (Fig. 11). This data suggests that the early Ca^2+^ mobilization event induced by LtxA binding, which is responsible for initiating the downstream cascade of events leading to cell death, may rely on activation of P2X_7_Rs. Understanding that ATP release from LtxA treated Jurkat cells occurs within seconds, it is possible that the release of ATP and its binding to P2X_7_R, and not intracellular Ca^2+^ mobilization, is the first event involved in the LtxA mediated cell death of Jurkat cells. If this is indeed the case, we would not be the first to demonstrate the ATP-induced propagation of calcium signals. Zumerle et al., for example, demonstrated in their work that triggered macrophages are able to release ATP and propagate calcium signals in untriggered bystander cells^42^. It has also been observed that binding of ATP to P2Y receptors increases inositol 1,4,5-triphosphate, releasing Ca^2+^ from the ER stores^43^. This released Ca^2+^ then goes on to activate single-membrane channels leading to further release of ATP and propagation of signals to neighboring cells^43^.

**Figure 12:**
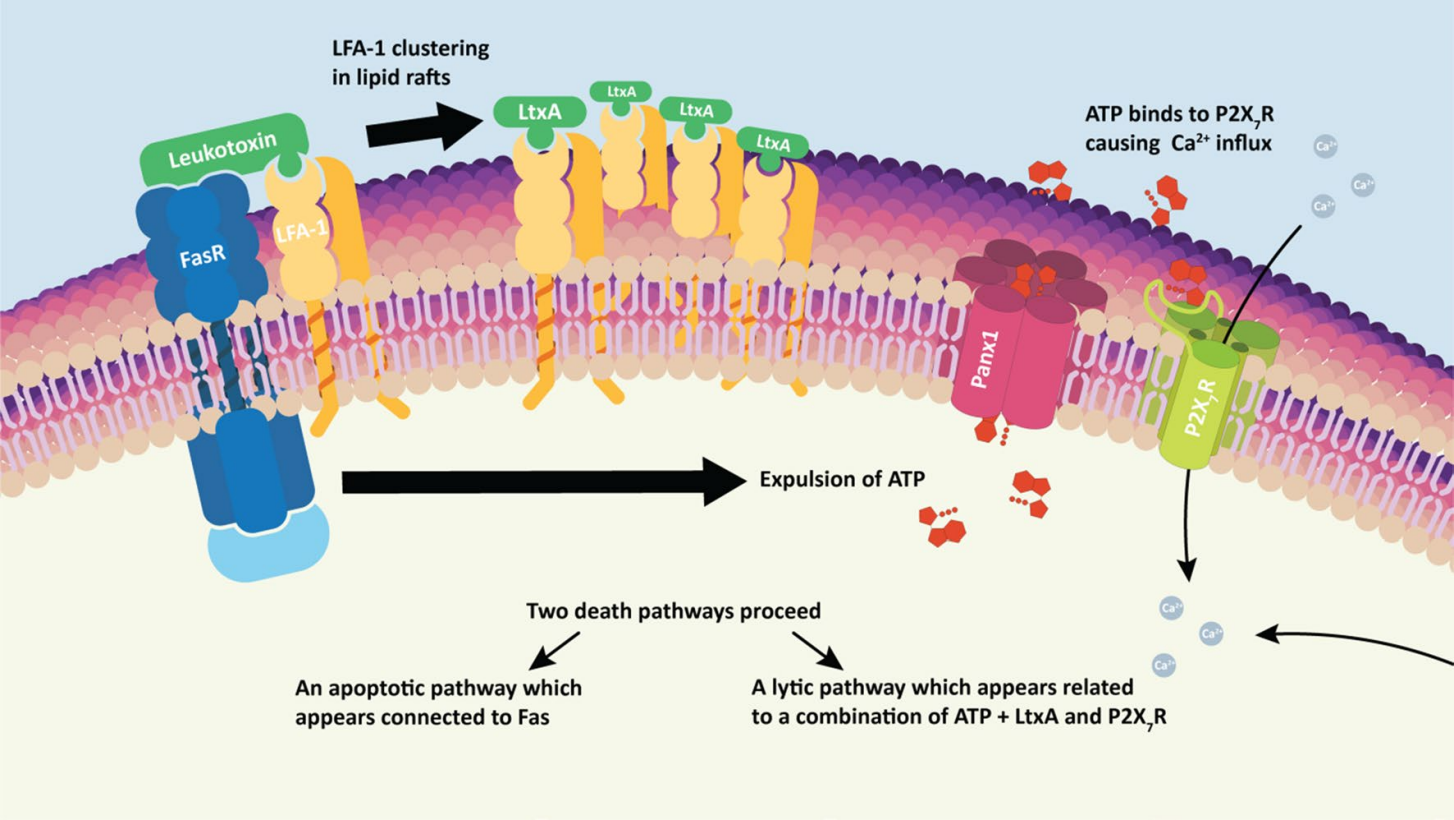
Proposed model. This model describes the proposed molecular sequence of events carried out during the LtxA mediated cell death of Jurkat cells. LtxA first interacts with susceptible cells by binding to LFA-1 which is believed to be coupled to the Fas death receptor. We show in this report that it appears that the next step following LtxA and membrane interaction is the controlled expulsion of ATP via Panx1 channels. The release of ATP then leads to intracellular Ca^2+^ mobilization, clustering of LFA-1 into lipid rafts, and subsequent cell death via apoptosis and necrosis. We additionally show that the elevations in intracellular Ca^2+^ appear to be dependent on ATPs interaction with the P2X_7_R.

Here we report that the release of ATP is most likely the first step involved in LtxA mediated Jurkat cell death. eATP leads to the autocrine/paracrine activation of P2X_7_ receptors which serves to propagate the mobilization of intracellular Ca^2+^, ultimately triggering the cascade of subsequent downstream events leading to cell death. For a summary of events, refer to the proposed model shown in Fig. 12.

## Materials and methods

### Cell lines and culture methods

Human white blood cell lines (Jurkat E6.1 and THP-1) were purchased from American Type Culture Collection [ATCC] (Manassas, VA). Knockout (K/O) cells deficient in Panx1 were generated using CRISPR/Cas9 gene editing methodology (necessary constructs and reagents were purchased from Invitrogen). All white blood cell lines were grown in Roswell Park Memorial Institute (RPMI) 1640 (Life Technologies) media supplemented with 10% heat inactivated (HI) fetal bovine serum (FBS; Life Technologies). In certain cases, K/O cells were supplemented with blasticidin and/or puromycin to select for K/O only cells. All cells were maintained in a cell culture incubator set to 37 °C and 5% CO_2_.

### Purification of LtxA

Leukotoxin was purified from cell culture supernatants of *A. actinomycetemcomitans* strain NJ4500. Additionally, an *A. actinomycetemcomitans* strain NJ4500 clone capable of growing in animal free media was isolated. LtxA purification procedures were developed and purified from culture supernatants at Paragon Biosciences (Baltimore, MD).

### Flow cytometry

#### Apoptosis (Annexin)

Cells (1 × 10^6^/mL) were treated with various concentrations of LtxA for indicated times. Following treatment, 100 µL of treated cells were transferred to 96-well black bottom plates. An aliquot of 100 µL of Nexin reagent (Millipore) was then added to each well containing 100 µL of cells. The plate was incubated in the dark and on a shaker for 10 min. Following incubation, the luminescence was read using a microtiter plate reader (BioTek Synergy 2). Treatments were performed in triplicate and 5000–10,000 events were recorded. Apoptosis was determined as the percentage of Annexin V^+^ cells relative to an untreated cell control.

### Measurement of ATP and extracellular ATP

ATP and extracellular ATP was measured via CellTiter-Glo, which catalyzes the oxidation of luciferin in the presence of ATP and produces luminescence. Cells (1 × 10^6^) in media were left untreated or treated with concentrations of LtxA or STS in 24-well plates. Following treatment, 100 µL of cells were transferred in triplicate into 96-well black wall and bottom plates and up to 100 µL of CellTiter-Glo reagent was added to each well. For extracellular ATP analysis, the cells were centrifuged at 250*g* for 5 min and 100 µL of the supernatant was added prior to the addition of CellTiter-Glo. The plate was mixed on an orbital shaker for 2 min. The plates were incubated at 10 min at room temperature. ATP (luminescence) was detected using a microtiter plate reader set to read the top of each well.

### Measurement of caspase activity

Caspase activity was measured via Caspase-Glo, where caspase cleavage of the proluciferin DEVD substrate, a substrate for luciferase (aminoluciferin) is released, and in the presence of luciferase and ATP, results in the production of luminesce. Cells (1 × 10^6^) in media were left untreated or treated with concentrations of LtxA or STS in 24-well plates. Following treatment, 100 µL of cells were transferred in triplicate into 96-well black wall and bottom plates and 50 µL of Caspase-Glo reagent was added to each well. Plates were incubated for 30 min at room temperature. Caspase activity (luminescence) was detected using a microtiter plate reader set to read the top of each well. The following Caspase-Glo assay systems were used for analysis: Caspase-Glo 3/7 assay (Cat. No. G8090). Caspase Glo 8 assay (Cat. No. G8200). Caspase Glo 9 assay (G8210).

### CytoTox-ONE homogenous membrane integrity assay

Jurkat cells were collected at 1 × 10^6^ cells/mL and treated with concentrations of LtxA at various time points. At the end of the treatment, 100 µL of each treatment sample was transferred to a 96-well plate in triplicate. For the positive control, 2 µL of lysis reagent was then added to the appropriate wells in triplicate. Equal amounts of assay reagent were added to each well and the plate was incubated at room temperature for 10 min. Stop solution (50 µL) was then added to each well and the plate was transferred to a shaker for 10 s. The plate was then read with an excitation wavelength of 560 nm and an emission wavelength of 590 nm. Cytotoxicity was calculated using the following equation:

$${\text{Percent Cytotoxicity}} = 100 \times ({\text{Experimental}} - {\text{Culture Medium Background}})/({\text{Maximum LDH Release}} - {\text{Culture Medium Background}})$$

### Cell depletion assay

Cell depletion assays were performed by counting cells with an automated cell counter (BIO-RAD, TC-20). Following treatment, wells containing cells were mixed with a pipette and 10 µL of cell suspension was transferred to an automated cell counting hemacytometer slide. The total cell count was recorded for three independent replicates and averaged. Percent depletion was calculated by dividing the treatment cells by the control cells and multiplying by 100.

### Western blot analysis

Cells (1 × 10^6^) in media were left untreated or treated with various concentrations of LtxA. The cells were collected and centrifuged at 250*g* for 5 min. The pellet was washed two times with 1 mL of sterile DPBS (HyClone Cat# SH30028.03). Following washing, pellets were resuspended with 100 µL of 1× lysis buffer (Cell Signaling Cat# 9803) diluted in sterile nuclease free water (Sigma Cat# W4502) and supplemented with protease and phosphatase inhibitor cocktail (Sigma Cat# PPC1010-1) at a ratio of 10 µL per 1 mL. The lysis buffer containing tubes were incubated at 4 °C for 30 min. After 30 min, 25 µL of NuPAGE LDS sample buffer (4× stock—Cat# NP0008) was added to the tube and the tube was passed through QIAshredder columns (Qiagen Ref# 79656) for 1 min at 20,000*g*. The flow through was collected and 5 µL of 2-Mercaptoethanol (Sigma Cat#M3148) was added. Each tube was then heated using a heat block set to 90 °C for 5 min. The lysate tubes were transferred to an ice bucket and 10 µL of lysate was loaded to each well of a precast NuPAGE Bis-4-12% Tris Protein Gel (Invitrogen Cat#NP0321) that was previously transferred to a X-Cell Surelock chamber containing 1 L of 1× NuPAGE MES SDS Running Buffer (Novex Ref#NP0002). To at least one well, 5 µL of Precision Plus Protein Dual Color Standards (BIORAD Cat#161-0374) protein ladder was added and run at 200 V for 30–35 min. The protein bands were transferred to a nitrocellulose membrane using the iBlot 2 NC Mini Top and Bottom Stack System as per the manufacturer’s instructions (Invitrogen Ref# IB23002). The assembled stack was then transferred to the iBlot 2 Dry Blotting System (Invitrogen Cat#IB21001) and the transfer was completed using the preprogrammed method recommended by the manufacturer: P0 (20 V for 1 min, 23 V for 4 min, 25 V for remainder—7-min run). The nitrocellulose membrane was removed from the assembly stack and kept wet with 1× wash buffer (PBS supplemented with 0.1% Tween 20) and then blocked with 5% nonfat dry milk prepared in 1× wash buffer for 1 h at room temperature on a shaker. The nitrocellulose membrane was then washed 3× for 5 min each with 1× wash buffer. The membrane was then probed with the appropriate antibody solution (1:1000) and left overnight on a shaker at 4 °C. The membrane was then washed 3× for 5 min each with 1× wash buffer and probed with the secondary antibody conjugated with HRP (1:1000) for 1 h on a shaker at room temperature. The membrane was again washed 3× for 5 min each with 1× wash buffer. The membrane was then developed using a fresh solution of approximately 5–6 mL of chemiluminescence substrate Clarity Western ECL Substrate (BIO-RAD Cat#170-5060). The protein bands were visualized using Molecular Imager ChemiDoc XRS+ Imaging System (BIO-RAD).

### CRISPR/Cas9 knockout generation

#### Generation of Cas9 expressing Jurkat cell line

Jurkat cells at a volume of 20 mL and concentration of 1 × 10^5^ cells/mL were prepared in RPMI supplemented with 10% HI FBS. Two sterile 15 mL conical tubes each containing 6 mL of the above cell suspension were supplemented with 8 µg/mL of polybrene (Santa Cruz Cat#SC-134220). Ready to use Cas9 LentiArray Lentivirus (Invitrogen; Cat# A32064) at a concentration of >10^7^ TU/mL (10^4^ TU/µL) was removed from a − 80 °C freezer and thawed until only small ice crystals remained. The lentiviral tube was then put on ice and 60 µL was added to one of the conical tubes to achieve a multiplicity of infection (MOI) of 1 (6 × 10^5^ lentiviral particles per 6 × 10^5^ cells). The other conical tube did not receive lentiviral treatment and served as a negative control. Both conical tubes were centrifuged at 800*g* at room temperature for 90 min to enhance surface binding and viral infectivity. Following centrifugation, the supernatant was carefully aspirated and the cells were resuspended with 6 mL of fresh medium and gently transferred to T-25 cell culture flasks. The flasks were then incubated at 37 °C/5% CO_2_ overnight (~18 h) without any disruption. The following day, the cells were gently transferred from the T-25 flask to a new 15 mL conical tube. The tubes were spun down at 200*g* for 5 min. The supernatant was aspirated and the cells were resuspended with 6 mL of fresh RPMI and transferred to a new T-25 flask. At 72 h post transduction, 10 µg/mL of blasticidin was added to the flasks and allowed to incubate with the cells for 7 days. Selection media was replaced every 3 days until the negative control cells were dead.

#### Single cell clone isolation

The Cas9 expressing Jurkat cells were kept incubating and allowed to further expand. Isolation of a single clone was achieved by performing the limiting dilution cloning (LDC) assay directly from the pool of stable transductants (i.e., Blasticidin-resistant cells). Blasticidin resistant cells were serially diluted to a final concentration of 5 cells/mL (0.5 cells/100 µL) using RPMI supplemented with 10 µg/mL of blasticidin. Using a multi-channel pipette, 100 µL of the cells were transferred into individual wells of a 96-well plate. The plates were incubated at 37 °C in a humidified 5% CO_2_ incubator and replaced with fresh blasticidin containing medium every 3–4 days. After 10–12 days of incubation, the wells were visually inspected under a microscope to identify which wells displayed evidence of colonies that derived from a single cell (monoclonal cells). Once identified, the monoclonal cells were transferred to a 24-well plate containing RPMI with blasticidin. The cells were incubated at 37 °C and scaled up every 2–5 days by transferring each clonal population into a larger plate or vessel (i.e., from 24-well plates to 6-well plates or T-25 flasks, and then to T-75 flasks). Once healthy and expanded clonal populations had been achieved, five vials from each clone were cryopreserved and assessed for cell morphology, growth kinetics, and Cas9 protein expression.

#### Generation of Pannexin-1 CRISPR/Cas9 knockout

A 24-well plate containing 5 × 10^4^ of Jurkat cells stably expressing Cas9 nuclease were expanded by incubating the cells at 37 °C/5% CO_2_. Transduction medium was prepared by adding sgRNA lentiviral particles (Dharmacon, Cat# VSGH10148-EG24145) to prewarmed RPMI at an MOI of 0.3. The cells were then further incubated at 37 °C/5% CO_2_ for 4–6 h. At 24–48 h post-transduction, the medium was replaced with selection medium (RPMI w/10% HI-FBS supplemented with 0.6 µg/mL of puromycin and 10 µg/mL of blasticidin). The selection medium was replaced every 2–3 days and the presence of dead cells was assessed daily. Once the cells were determined to be growing normally in selection medium, they were expanded, and aliquots were prepared for cryopreservation. Single cell clonal expansion was again performed. Western blots were performed to determine which knockout resulted in the highest knock down efficiency.

### Calcium (Ca^2+^) mobilization

Cells were treated in a 24-well plate and then centrifuged at 430*g* for 5 min. The cells were then suspended in 100 µL of fluo-8 dye loading solution and transferred to a 96-well poly-d-lysin plate (Abcam ab112129). The plate was immediately centrifuged at 120*g* for 2 min and incubated at 37 °C/5% CO_2_ for 30 min and then incubated at room temperature for an additional 30 min. The calcium flux assay was performed by monitoring the fluorescence intensity at Ex/Em = 490/525 nm.

### Statistical analysis

Bars on graphs represent the means from at least three independent experiments and error bars represent the standard error of the mean (SEM). Standard error was calculated as standard deviation/sq rt (sample size). For statistical analyses, data were subjected to an unpaired 2-tailed Student’s *t* test, with p ≤ 0.05 considered to be statistically significant. All calculations were performed using GraphPad Prism Software. *p ≤ 0.05, **p ≤ 0.01, ***p ≤ 0.001.

## Supplementary Information

Supplementary Figures.

## Data Availability

The datasets generated during and/or analyzed during the current study are available from the corresponding author on reasonable request.
