# Supplementary material for: Leukotoxin (LtxA/Leukothera) induces ATP expulsion via pannexin-1 channels and subsequent cell death in malignant lymphocytes
Source: Sci Rep. 2021 Sep 10;11:18086. doi: 10.1038/s41598-021-97545-4 (PMC8433231; doi:10.1038/s41598-021-97545-4)
Supplement: Supplementary file 1 — Supplementary Figures. [file 41598_2021_97545_MOESM1_ESM.docx]

**Leukotoxin (LtxA/Leukothera) induces ATP expulsion via pannexin-1 channels and subsequent cell death in malignant lymphocytes**

Derek J. Prince^1^, Deendayal Patel^2^ and Scott C. Kachlany^1,*^

^1^Department of Oral Biology, Rutgers School of Dental Medicine, Newark, NJ 07103, United States

^2^Gibraltar Laboratories, Inc., Fairfield, NJ, 07004

^*^Address correspondence to: Scott C. Kachlany; kachlasc@rutgers.edu

Running Title: Leukotoxin mediated ATP expulsion

Supplementary Figure S1


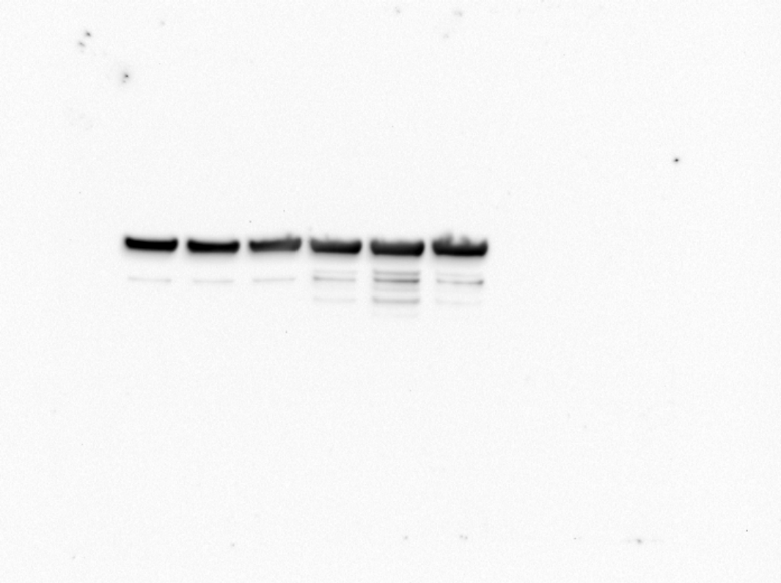


**β-actin**

**Jurkat THP**

MW (kDa)

45

Full uncropped blot of Figure 3 showing β-actin loading control at 45 kDa.

Supplementary Figure S2


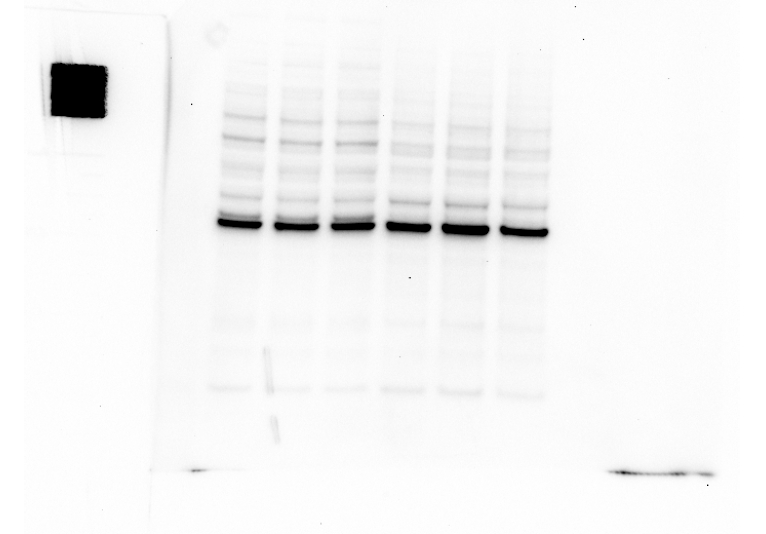


**P2X_7_R**

38

MW (kDa)

**Jurkat THP**

Full uncropped blot of Figure 3 showing P2X_7_R staining.

Supplementary Figure S3

MW (kDa)


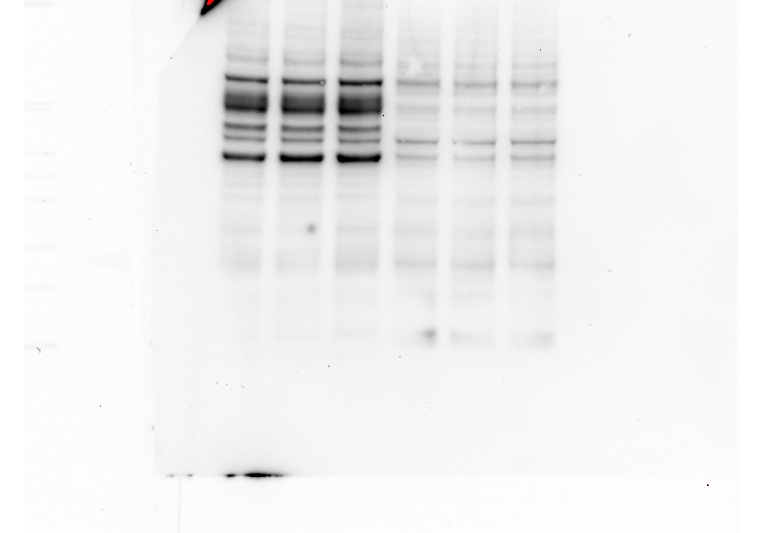


**Cleaved Panx1**

18

52

45

**Jurkat THP**

Full uncropped blot of Figure 3 showing cleaved Panx-1 staining at 18 kDa.

Supplementary Figure S4

MW (kDa)


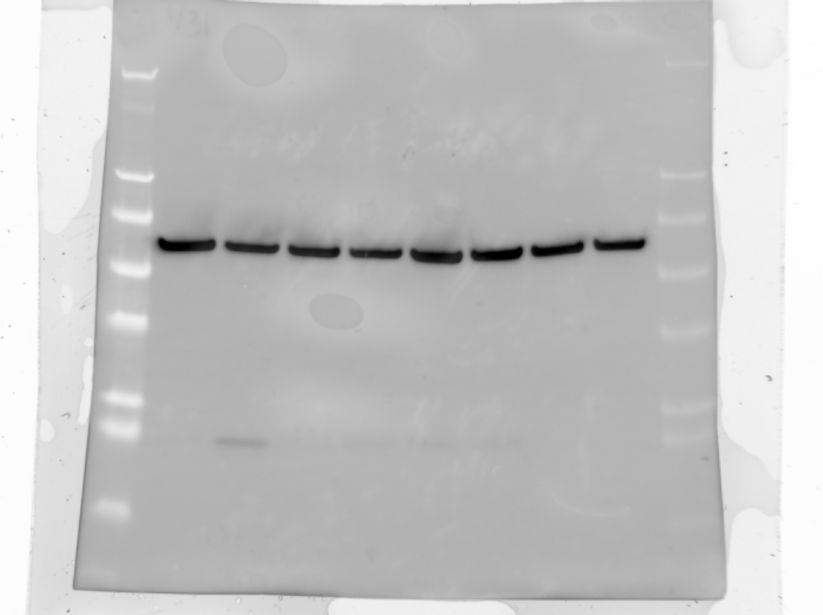


198

62

**β-actin**

49

38

28

18

14

6

Full uncropped blot of Figure 3 showing β-actin loading control at 45 kDa.

Supplementary Figure S5

A)


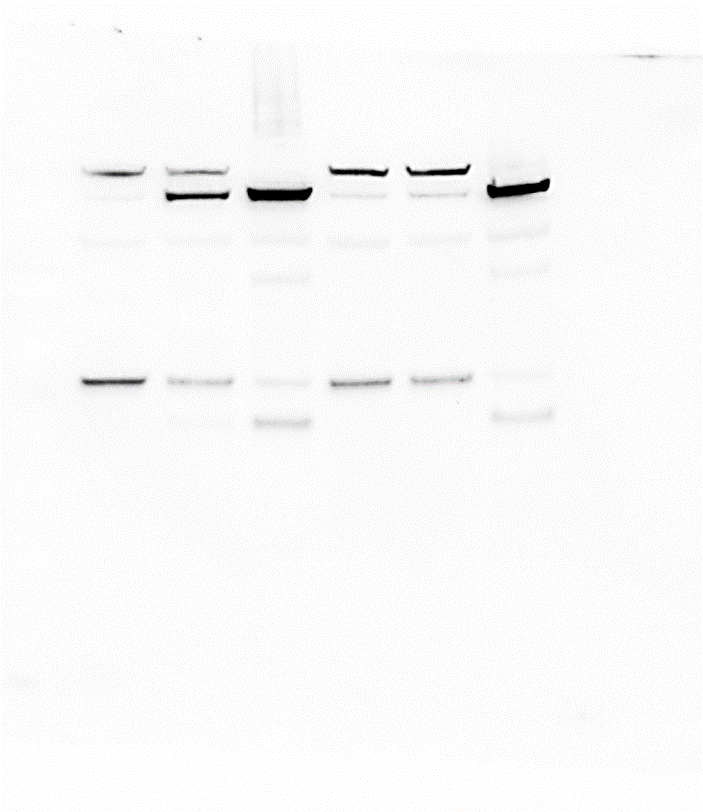


116

89

MW (kDa)

B)


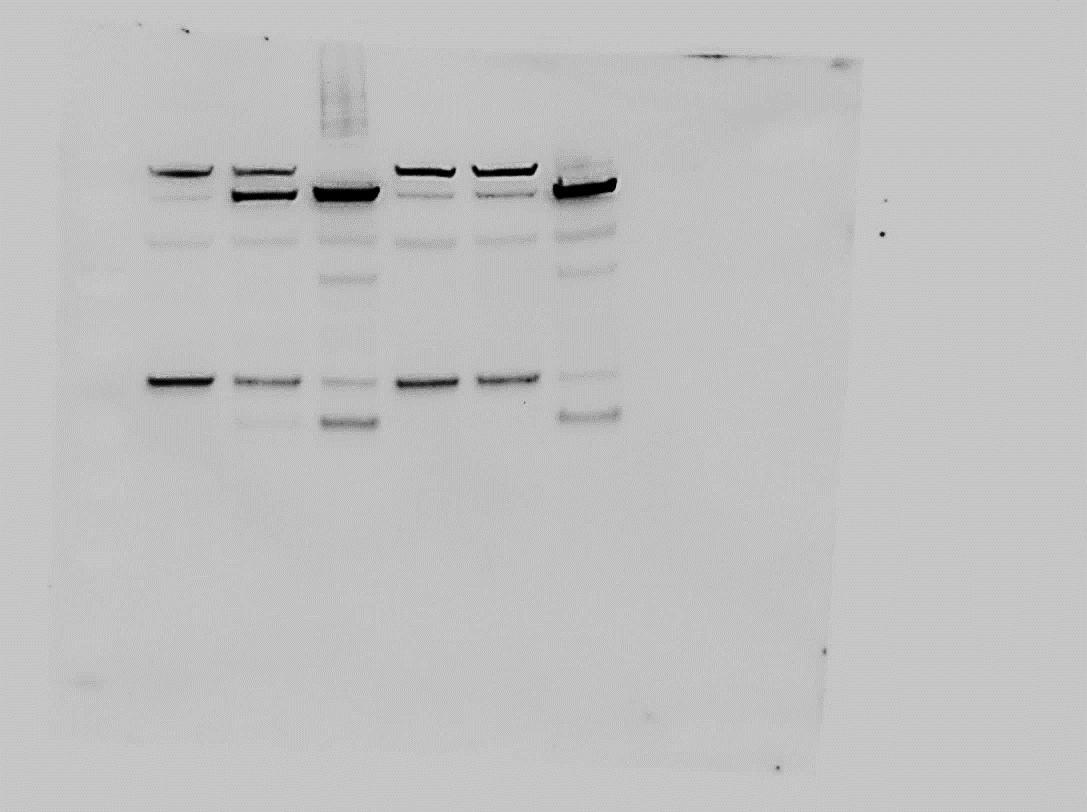


C)


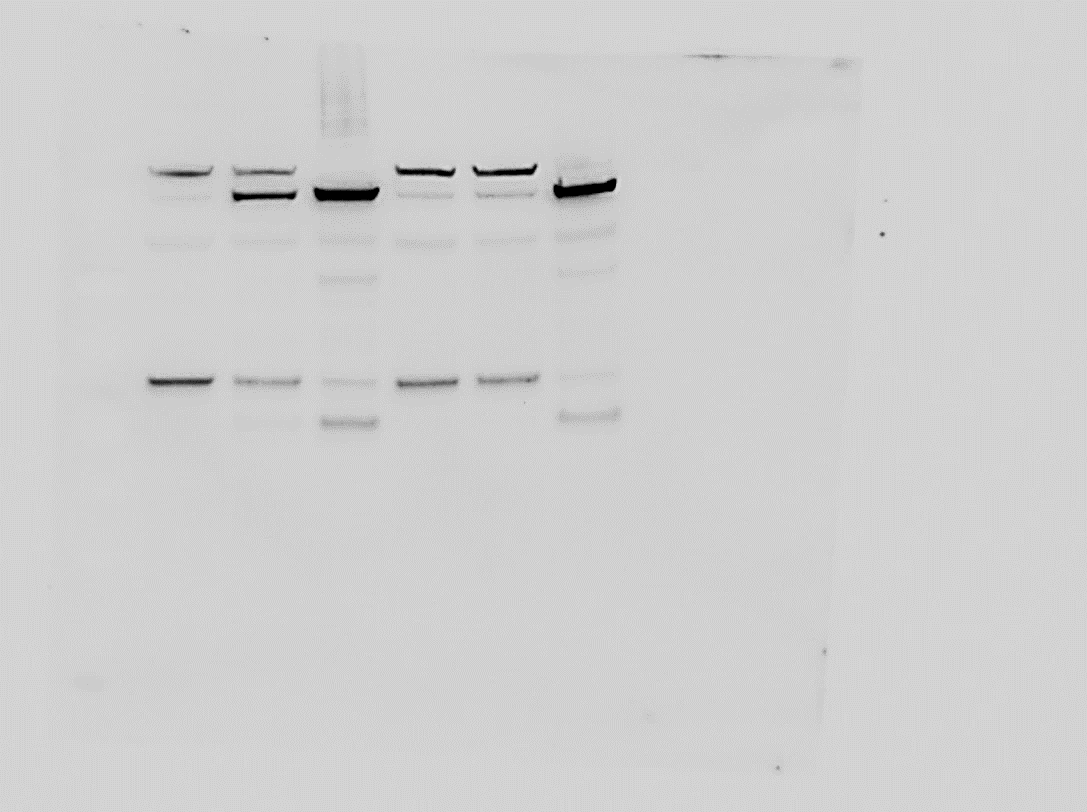


D)


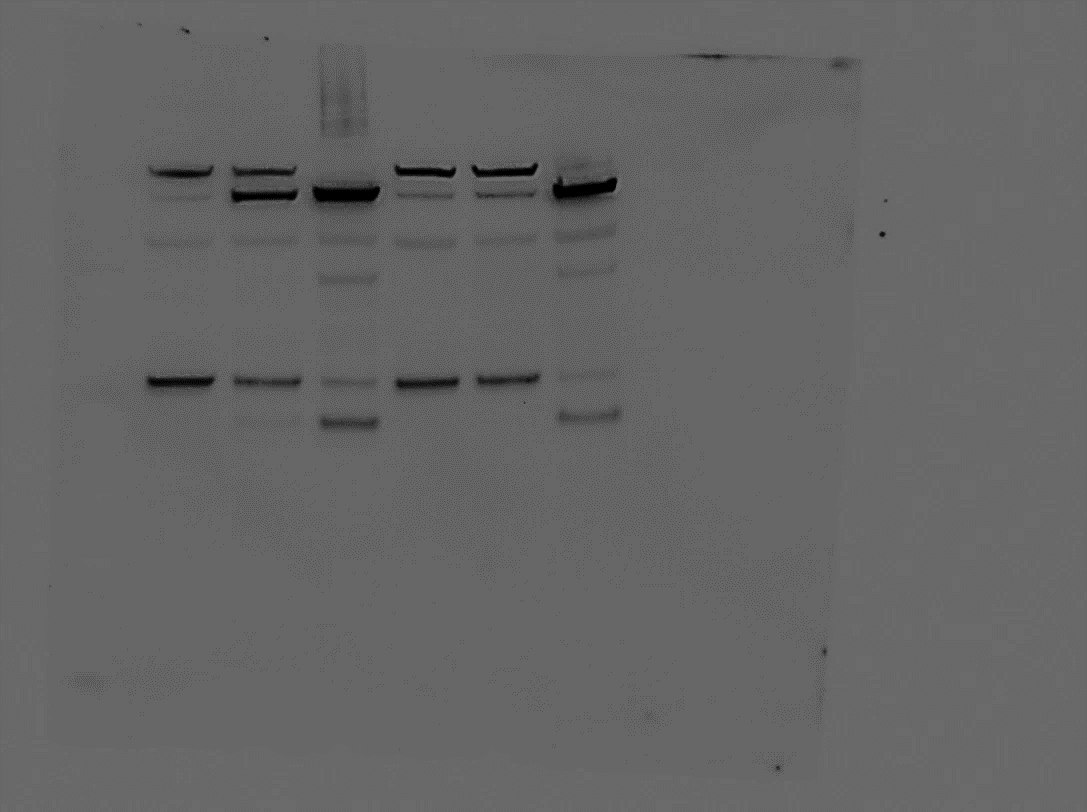


Full uncropped blot of Figure 8 showing full length PARP (116 kDA) and cleaved PARP (89 kDa).
